# A Technology-Based Intervention Among Young Men Who Have Sex With Men and Nonbinary People (The Conectad@s Project): Protocol for A Vanguard Mixed Methods Study

**DOI:** 10.2196/34885

**Published:** 2022-01-13

**Authors:** Thiago Silva Torres, Emilia Moreira Jalil, Lara Esteves Coelho, Daniel Rodrigues Barros Bezerra, Cristina Moreira Jalil, Brenda Hoagland, Sandra Wagner Cardoso, Sean Arayasirikul, Valdilea Gonçalves Veloso, Erin C Wilson, Willi McFarland, Beatriz Grinsztejn

**Affiliations:** 1 Instituto Nacional de Infectologia Evandro Chagas, Fundação Oswaldo Cruz Rio de Janeiro Brazil; 2 Center for Public Health Research, San Francisco Department of Public Health San Francisco, CA United States; 3 Department of Epidemiology and Biostatistics, University of California San Francisco San Francisco, CA United States

**Keywords:** sexual and gender minorities, young MSM, Brazil, HIV prevention, technology-based adherence intervention, HIV

## Abstract

**Background:**

In many parts of the world, including Brazil, uptake for biomedical interventions has been insufficient to reverse the HIV epidemic among key populations at high risk for HIV, including men who have sex with men. Young MSM (YMSM), particularly Black YMSM, have high HIV incidence, low viral suppression, and low preexposure prophylaxis (PrEP) uptake and adherence. Therefore, novel approaches to increase the HIV biomedical interventions uptake by YMSM are urgently needed.

**Objective:**

We describe the Conectad@s Project, which aims to: (1) estimate the prevalence and incidence of HIV and other sexually transmitted infections, the onset of sexual risk behavior, and barriers to biomedical interventions among YMSM aged 18 to 24 years in Rio de Janeiro, Brazil; and (2) conduct a technology-based adherence intervention study to promote a rapid linkage of YMSM to HIV care or prevention, and support and sustain adherence.

**Methods:**

A cross-sectional survey will be conducted with 400 YMSM recruited using respondent-driven sampling (RDS) adapted for social media-based sampling, preceded by a formative phase. HIV and sexually transmitted infections testing will be conducted, including early HIV infection biomarker detection. Behavioral, partnership, network, and structural measures will be collected through structured questionnaires. All individuals recruited for the survey will have access to HIV risk assessment, antiretroviral therapy (ART), PrEP, prevention counseling, and a technology-based adherence intervention. Those who accept the adherence intervention will receive weekly text messages via a social networking app (WhatsApp) for 24 weeks, with follow-up data collected over 48 weeks.

**Results:**

The Conectad@s project has been approved by our local institutional review board (#CAAE 26086719.0.0000.4262) in accordance with all applicable regulations. Questionnaires for the RDS survey and intervention were developed and tested in 2020, formative interviews were conducted in January and February 2021 to guide the development of the RDS, and enrollment is planned to begin in early 2022.

**Conclusions:**

The Conectad@s Project is a vanguard study that, for the first time, will apply digital RDS to sample and recruit YMSM in Brazil and rapidly connect them to ART, PrEP, or prevention counseling through a technology-based adherence intervention. RDS will allow us to estimate HIV prevalence among YMSM and measure HIV infection biomarkers in the context of the onset of risky behavior. The data will lay the groundwork to adapt and implement HIV prevention strategies, identify barriers to the earliest HIV infection diagnosis, immediate ART or PrEP initiation, and detect new clusters of HIV transmission.

**International Registered Report Identifier (IRRID):**

DERR1-10.2196/34885

## Introduction

Reported HIV cases in Brazil are increasing among gay, bisexual, and other men who have sex with men (MSM), particularly among the youngest, with a considerable disparity of infection for this population [1,2]. In 2020, 53% of reported male HIV cases were MSM [1]. Reported cases in Brazilian surveillance may be incomplete as MSM status is likely under-reported among men classified as heterosexual (30% of male cases) and those with unknown risk (14% of male cases) due to stigma [3,4]. National surveillance data show a 7-fold increase in the rate of HIV cases reported among Brazilian men aged 15-24 years between 2009 and 2019 [1]. A population-based survey in selected capital cities in Brazil found 18% HIV prevalence in MSM in 2016 [5], increasing from 12% seven years earlier [6]. In addition, MSM surveyed in 2016 were notably younger than those in 2009, while HIV prevalence rose [7].

Brazil was the first low-income/middle-income country to provide free antiretroviral therapy (ART) for HIV treatment, participate in clinical trials proving preexposure prophylaxis (PrEP) efficacy [8] and PrEP demonstration projects [9-12], and establish a national policy to provide PrEP at no cost within the National Public Health System (SUS). Nevertheless, uptake of these biomedical interventions has been insufficient to reverse or even slow the HIV epidemic among young MSM (YMSM).

Data from a national web-based survey of Brazilian MSM indicated higher HIV risk and lower use of biomedical prevention among YMSM aged 18-24 years compared to older MSM, including condomless anal sex, being unaware of PrEP, never testing for HIV, and not using PrEP [13]. Among 16,667 Brazilian MSM recruited in web-based studies, YMSM showed increased odds of binge drinking and condomless receptive anal sex and decreased chances of high perceived HIV risk [14]. Youth also had an increased probability of high-risk behaviors measured by the HIV Incidence Risk Index, even when adjusted by race, income, education, sexual orientation, steady partner, previous sexually transmitted infection (STI), and ever testing for HIV [15].

The data point to disparities for Black Brazilians, including late HIV diagnosis, not being on ART, low virological suppression rates, and low PrEP adherence [9,16]. Black Brazilians had over 50% increased odds of experiencing discrimination than White individuals, even after controlling for income, education, social status, and health problems [17]. In surveillance data, the proportion of HIV cases for Black and *Pardo* (mixed-Black) Brazilians rose from 51% to 63% from 2009 to 2019 [1]. MSM populations may face various forms of stigma, including internalized, perceived, experienced, and layered stigmas [18], and Black*/Pardo* MSM populations also face structural racism, which may increase their vulnerability to HIV infection in comparison to White MSM.

The Rio de Janeiro metropolitan area, with 13 million inhabitants and 22 municipalities, is the second-largest in the country and the 16th largest urban area in the world. Rio de Janeiro state accounts for 10% of HIV cases nationwide, 90% of them residing in the metropolitan area [1], where mortality due to HIV-related causes, particularly tuberculosis, remains persistently above the Brazilian mean [1]. The state also demonstrates high rates of late-stage HIV and death, which suggest poor engagement along the HIV care continuum, including late diagnosis, low ART use, and insufficient virologic suppression [1]. Emerging evidence points to resurging HIV among YMSM, including high case detection rates at HIV testing sites [19]. HIV prevalence among YMSM aged 18-24 years in Rio de Janeiro increased from 4.4% to 13.3% between 2009 and 2016 [20]. Drivers of HIV infection among Brazilian YMSM, likely to intersect in Rio de Janeiro, remain understudied and unaddressed. Therefore, we designed the Conectad@s Project, a respondent-driven sampling (RDS)-based study to specifically reach and engage YMSM in Rio de Janeiro, Brazil. Our primary aims are: (1) to estimate the prevalence and incidence of HIV and other STIs, the onset of risky behavior, and barriers to biomedical interventions among YMSM aged 18 to 24 years, and (2) to conduct a technology-based adherence intervention study to promote a rapid linkage of YMSM to HIV care or prevention.

## Methods

The institutional review board of the INI-Fiocruz reviewed and approved this protocol on February 27, 2020 (#CAAE 26086719.0.0000.4262) in accordance with all applicable regulations.

### Study Design

RDS has gathered robust samples of MSM in studies worldwide, including in Rio de Janeiro [5,6]. To reach and engage YMSM in Rio de Janeiro, Brazil, we will conduct an RDS-based study at the National Institute of Infectious Diseases Evandro Chagas (INI)-Fiocruz. The RDS will be adapted to include social media-based methods. The approach builds on research on young transgender women aged 15-24 years in San Francisco, United States [21]. Starting with initial “seeds” (ie, initial participants who start recruitment chains), successive waves of referrals will reach diverse social networks of YMSM. We anticipate that peer referrals through social media connections will particularly appeal to Brazilian YMSM who spend much of their time on mobile-based apps (eg, WhatsApp) and digital social media. Despite the discussion around the generalization of their data, RDS-based studies provide estimates of HIV risk among hard-to-reach populations [22] and allow the recruitment of these populations into longitudinal studies (ie, observational or intervention). Subsequently, we will offer all recruited individuals a prospective 48-week technology-based adherence intervention study to promote a rapid linkage of YMSM to HIV care or prevention and support and sustain adherence.

### Eligibility Criteria

Individuals will be included if they (1) self-identify as men (cis or trans) or gender nonbinary, (2) report ever having engaged in anal sex with men or gender nonbinary persons with a penis, (3) are aged 18 to 24 years, (4) reside or spend most of the time in Rio de Janeiro metropolitan area, (5) did not previously participate in the study, and (6) possess an electronic referral coupon from a peer acquaintance participant. In this study, we collectively refer YMSM to young (18-24 years) cisgender men, transgender men, and non-binary or gender-nonconforming individuals. Exclusion criteria include individuals who self-identify as women (cis or trans).

### Formative Phase

RDS-based studies require a formative phase to verify theoretical assumptions and identify logistical constraints and solutions [23]. These objectives are met through focus group discussions, key informant interviews, and pilot testing. The theoretical RDS assumptions are: (1) members of the population know each other as members, (2) social networks are interconnected within a few degrees of separation, (3) sampling occurs with replacement, (4) network size is reported accurately, and (5) people recruit approximately randomly from their network. RDS also depends on sufficient network density to make long recruitment chains [24]. Furthermore, the characteristics of social networks guide the selection of the number and type of “seeds.” The formative phase also identifies potential “bottlenecks” (ie, social and physical barriers between networks) and variables to track “equilibrium” (ie, stability in sample composition as the chains grow). Other logistical questions include study site acceptability, transportation, online and social media apps for electronic referrals, safety, confidentiality, and appropriate incentives for participation and recruiting peers. In addition, the formative phase will inform the development and pilot testing of the questionnaire, as well as the refinement of the technology-based intervention.

The formative phase has already been conducted, comprised of two focus group discussions (aged 18-19 and 20-24 years) of up to 10 participants each and individual interviews with up to 20 YMSM. Group discussions and individual interviews lasted 1-2 hours. YMSM were referred from HIV clinics, LGBTQIA+ (lesbian, gay, bisexual, transgender, queer/questioning, intersex, and asexual/aromantic/agender) nongovernmental organizations, and peer counselors at Instituto Nacional de Infectologia Evandro Chagas, Fundação Oswaldo Cruz (INI-Fiocruz). We used a maximum variation sampling approach to include a diversity of participants by place of residence, race/ethnicity, and age [25]. Group discussions and individual interviews were audio-recorded with field notes captured by the interviewer. Files were transcribed in Portuguese and translated into English. Content analysis using transcribed content is underway and will focus on RDS theory, logistics, and strategies to improve the study. The study team will analyze qualitative data and discuss the preliminary results related to the density of the social network and logistics to reach a consensus on strategies to improve the implementation of RDS in this study. If social networks appear weak or diffuse, we will develop an additional seed recruitment plan and alternative approaches to improve recruitment based on the findings within a theoretical probability-based sampling framework. Questions raised during the formative phase will be incorporated into the RDS study instruments to be quantified. A chosen subset of participants (approximately10) will pilot the questionnaire. Participants will provide feedback regarding comprehensibility, missing and unnecessary constructs, wording, and timing.

### Sample Size and Power Calculation

Within an acceptable margin of error, the sample size of RDS surveys is powered to measure key indicators of HIV risk and prevention in the population of YMSM (eg, HIV prevalence, PrEP awareness, willingness to use PrEP, and never testing for HIV). Assuming a design effect of 2.0 (typical for RDS) [26] and a 95% CI, 400 participants are sufficient to measure RDS-adjusted estimates within SD 5% over a wide range of point estimates (ie, 5%-40%). For example, 400 participants could measure HIV prevalence at 5%, SD 2.1% or being unaware of PrEP at 39%, SD 4.7%. A sample size of 400 also provides 80% power at a 95% CI to detect significant odds ratios for effect sizes of 1.9 or greater for key outcomes (eg, HIV infection, PrEP awareness, and willingness to use PrEP) and predictor variables (eg, race/ethnicity, perceived HIV stigma, and family support).

### Recruitment Methods

**Figure 1:**
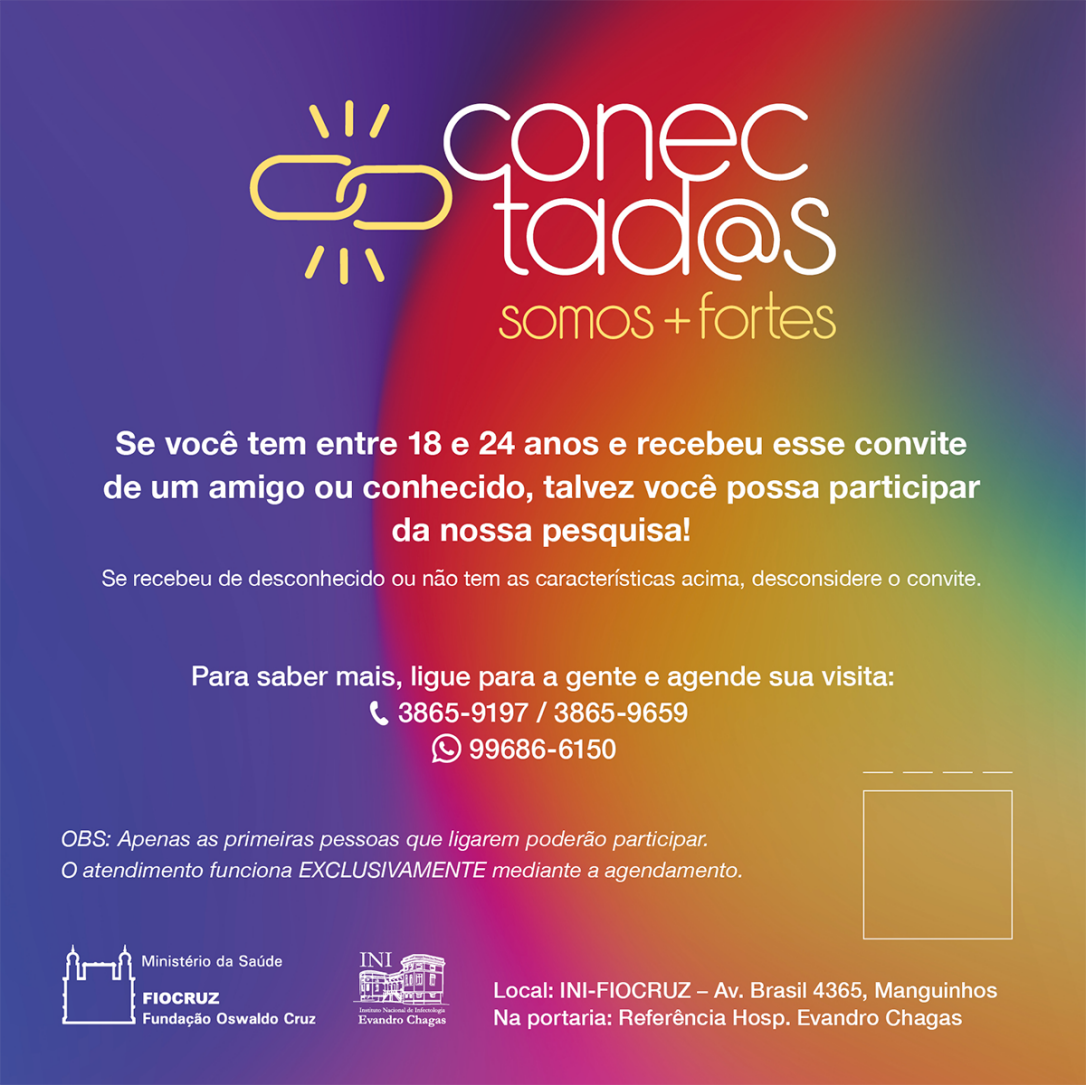
Digital coupon for the Conectad@s Project recruitment. Legend: Coupon used by seeds to recruit peers to the project, with the name (Conectad@s) followed by “we are strong,” inclusion criteria (18-24 years) and invitation to participate in the project only if coupon was received from a friend or acquaintance. Phone number and WhatsApp contact information to schedule an appointment are provided with a statement that scheduling is required.

RDS sampling begins similarly to “snowball sampling” (ie, with purposively selected “seeds” from diverse social networks who then refer peers to the study). Peer recruitment on RDS differs from snowball sampling on key procedural and theoretical factors that enable better estimates of disease prevalence [22]. Seeds will be chosen for their connections to other YMSM, enthusiasm for the research, and belonging to different social circles. As with all other participants, the seeds must be eligible and undergo all study procedures. After completion, the seeds will be trained to recruit their peers using a digital coupon. This process will create a link between the recruiter and recruits, providing information needed for statistical adjustment. Digital coupons (Figure 1) will be shared on mobile apps and web-based texting platforms [25,27]. Digital referrals significantly increased recruitment of a cohort of transwomen aged 15-24 years in San Francisco, United States [21,25]. Upon presentation of a digital coupon to the study site, recruits will undergo similar procedures if eligible. The process will continue until the sample stabilizes on key characteristics (“equilibrium”) and the sample size is met. RDS requires monitoring for “bottlenecks” (eg, chains that do not cross networks) and valid connection (eg, true acquaintances). The limited number of recruits per participant (typically 3) and incentives drive the propagation of long chains of referrals within and across social networks. Participants receive a “primary incentive” for their enrollment and a “secondary incentive” for each eligible participant they recruit. The value and type of incentives will be explored and determined in the formative phase.

### HIV Diagnosis Algorithm

All participants will be tested for HIV using an HIV rapid test and antibody/antigen 4th generation serology. Participants reporting recent anal condomless sex (<30 days) with a negative HIV rapid test will be screened for acute HIV infection through HIV RNA viral load (VL) testing.

### HIV Recency And Phylogenetic Testing

We will also test for recent HIV infection with the limiting avidity assay [28] to identify seroconversions occurring in the last few months. Recency data can help to identify possible transmission clusters and permit the calculation of HIV incidence by providing the timing of seroconversion [28]. The detection of acute infections may contribute to identifying active clusters of HIV transmission. HIV VL reaches high levels in the short period after infection, increasing the risk of HIV transmission. Recent HIV acquisition events will allow the characterization of drivers of new infections and avoid recall bias because remembering sexual partners once in the last few weeks is easier than over a longer time.

We will perform HIV phylogenetic testing on specimens with detectable HIV VL. Phylogenetic data will identify HIV strain types, ART resistance mutations, and clusters of transmission.

### STI Testing

The incorporation of biomarkers for acute and recent infection, as well as STIs, may be particularly valuable among YMSM, where the onset of behaviors leading to infection is more recent. We will evaluate STIs as markers of HIV sexual risk and causes of morbidity using nucleic acid amplification tests for oropharyngeal, urethral and rectal *Neisseria gonorrhea* and *Chlamydia trachomatis* (nontreponemal syphilis testing and if positive treponemal test), chronic hepatitis B (anti-HBs antibody, Hbs antigen, and total anti-HBc antibodies), chronic hepatitis C (anti-HCV), human papillomavirus testing and anal cytology. Participants diagnosed with STI will receive treatment according to Brazilian guidelines [29]. Participants screened positive for hepatitis B and C will undergo a complete diagnosis algorithm (ie, HCV viral load) [30] and will be referred to treatment. Participants without hepatitis B diagnosis and who have not been vaccinated will be referred for hepatitis B vaccination.

### Behavioral, Partnership, Network, and Structural Measures

Trained staff will administer face-to-face questionnaires to participants using tablets. Due to the COVID-19 pandemic, all efforts will be undertaken to minimize SARS-CoV-2 transmission. Before study procedures, participants will be screened for COVID-19 symptoms, and visits will consider the safety of all participants and the study team. Our instrument will build upon questionnaires previously used for MSM in Brazil and the United States. Table 1 presents examples of measurements, hypotheses, and sources of questionnaires.

Biological data, particularly markers of recent HIV and STI infection, will permit closer linkage to the events, behaviors, partnerships, and social and structural conditions that lead to HIV acquisition. We will also identify whether there are infection clusters within specific racial/ethnic groups, as previously seen among Black MSM in the United States [31]. With robust biomedical markers of infection, we will be able to discern whether tight sexual networks constrained by racial bias and discrimination impact the spread of HIV among Black and *Pardo* Brazilians and whether this is a recent phenomenon or ongoing over the course of the Brazilian MSM epidemic.

**Table 1:** Behavioral, partnership, network, and structural measures for the RDS^a^-based study.

Timeframe	Level/domain	Examples of measures/hypotheses
Acute infection: within the last several weeks	Individual	Events of likely acquisition and transmission; missed opportunities for prevention; STI^b^ co-infection.
	Partnership	Characteristics of most recent sexual partners (HIV status, disclosure, on ART^c^, PrEP^d^, and age) [32]; phylogenetic linkage.
Recent infection: within last 130-180 days	Individual	Missed opportunities for prevention; untreated STIs; HIV care cascade.
	Sexual network, with a focus on Black/*Pardo* Brazilians	Characteristics of recent sexual partners; age and racial mixing, with a specific focus on sexual networks of Black/*Pardo* Brazilians [31]; phylogenetic clustering; partner concurrency.
Long-standing infection: since the onset of risk or last HIV test	Previously undiagnosed	Missed opportunities for testing, linkage to care, experiences, and attitudes towards care providers.
	Previously diagnosed	Barriers to care, ART initiation, adherence [33-35], viral suppression; care cascade; care self-efficacy; perceived HIV stigma [36].
All (including and regardless of HIV status): since the onset of risk	Demographic	Racial disparities in risk, prevention, and care access (eg, race/ethnicity and socioeconomic status); homelessness, runaway; incarceration; digital health information.
	Risk and prevention	Onset of sexual risk, lifetime risk; substance use and chemsex [37]; HIV testing; internet use for sex (eg, apps); PrEP awareness, willingness, use [10,12,13,38]; knowledge and willingness to use new prevention technologies [39]; HIV perceived risk [40]; knowledge of HIV [41,42].
	Psychosocial, structural	Mental health [43,44], psychological distress, trauma [45], suicidality, and social support; testing, PrEP norms, and stigma [12]; peer support; family support; sexual orientation disclosure; experiences of discrimination [46,47], internalized homonegativity [48]; sexual compulsivity [49]; COVID-19 pandemic impact in personal life [50].
	Health and social welfare systems	Care-seeking and medical mistrust; health care participation; avoidance of care; perceived care access; “*Bolsa Familia*” (family grant or family stipend) program and other cash transfer programs; SUS^e^ experiences; food insecurity [51]; political context; digital health experiences.

^a^RDS: respondent-driven sampling.

^b^STI: sexually transmitted infection.

^c^ART: antiretroviral therapy.

^d^PrEP: preexposure prophylaxis.

^e^SUS: Sistema Único de Saúde (Brazilian National Public Health System).

### Technology-Based Adherence Intervention

All RDS participants will be invited to participate in a technology-based adherence intervention study. The intervention builds upon Health eNav, a text messaging HIV care linkage and retention intervention for young people living with HIV in San Francisco, United States, using an SMS-based platform to support ART adherence and provide HIV prevention counseling [21]. Based on a prior study, we anticipate 13% HIV prevalence [20] (N=52 individuals), 26 (50%) of whom will be newly diagnosed and 26 (50%) previously diagnosed (regardless of linkage to care). Therefore, we estimate that 65% (226/348) of HIV-negative YMSM, as previously described [15], will be interested and eligible for PrEP according to the Brazilian guidelines (eg, condomless anal sex, sex with HIV positive partner, and STI diagnosis in the last 6 months) [52].

HIV-negative participants enrolled in the intervention study will receive same-day PrEP, according to the Brazilian recommendations, plus a complete prevention package, including HIV/STI testing and treatment, counseling for HIV/STI risk reduction, hepatitis B vaccination, and condoms/lubricants distribution (Group 1: HIV-negative on PrEP). HIV-negative YMSM not eligible for PrEP or who do not accept PrEP may also be enrolled in the intervention study to receive the prevention package (including PEP, when indicated), risk assessments, and counseling (Group 2: HIV-negative not on PrEP).

HIV-positive YMSM enrolled in the intervention study will initiate same-day ART treatment according to Brazilian recommendations and will be further linked to care at the INI-Fiocruz HIV clinic (Group 3: HIV-positive on ART). HIV-positive YMSM previously diagnosed and who initiated ART prior to inclusion will also be invited to the intervention.

All participants enrolled will receive weekly reminders for 24 weeks via WhatsApp, the most common SMS platform in Brazil. Automatic messages will include scheduled medication reminders, if applicable, and posts. Periodic feedback from participants will adapt and improve the approach. (eg, adjustments or modifications on the content of electronic messages). The weekly reminders will be personalized. Participants will be able to select the timing of reminders and choose between having explicit medication reminders or factoid messages (not specific to medication), such as LGBTQIA+ community events, health facts, or a combination of both types of messages. All messages sent will solicit a response, so we will have backend data (eg, time, date, message content, etc) on whether the message was received and reviewed by participants. For example, we may send a medication reminder text, such as “Did you take your medication today?” Responses will be recorded in our database, but more importantly, we will know whether the participant was exposed to the text message regardless of receiving a formal response.

After enrollment, follow-up visits will occur at 4 and 12 weeks and then quarterly (24, 36, and 48 weeks). Briefly, during follow-up visits, we will collect information on study outcomes, such as HIV seroconversion, PrEP continuum endpoints, and PrEP adherence (using dried blood spots for TFV-diphosphate concentration among HIV-negative participants); HIV viral load suppression among HIV-positive participants; and STI incidence among all participants. The intervention study baseline visit procedures are the same as those conducted during the RDS visit. A detailed schedule of events and procedures is depicted in Table 2.

**Table 2:** Schedule of events for the Conectad@s Project.

			RDS^a^/ Baseline		Week 4		Week 12		Week 24		Week 36		Week 48		Early Termination^b^		HIV seroconversion visit		Unscheduled visit
**Questionnaires**																			
	Behavioral, partnership, network, and structural measures	X		X		X		X		X		X		X		—^c^		—	
	Technology-based intervention: inclusion	X		—		—		—		—		—		—		—		—	
	Technology-based intervention: acceptability	—		—		—		X		—		—		—		—		—	
**Laboratory procedures**																			
	HIV antigen rapid test	X		X^d^		X^d^		X^d^		X^d^		X^d^		X^d^		X		X^e^	
	HIV RNAPool	X^f^		X^f^		X^f^		X^f^		X^f^		X^f^		X^f^				X^f^	
	HIV RNA viral load	X^g^		X^h^		X^h^		X^g^		X^h^		X^g^		X^g^		X		X^e^	
	CD4/CD8	X^i^		X^j^		X^j^		X^i^		X^j^		X^i^		X^i^		X		X^e^	
	HIV recency testing	X^i^		—		—		—		—		—		—		—		X^e^	
	HIV genotyping	X^k^		X^l^		—		—		—		—		—		X		X^e^	
	Urine (CT^m^/NG^n^)	X		—		—		X		—		X		X		X		—	
	Oropharyngeal swab (CT/NG)	X		—		—		X		—		X		X		X		—	
	Anal swab (CT/NG)	X		—		—		X		—		X		X		X		—	
	Anal swab (HPV^o^)	X		—		—		—		—		—		—		—		—	
	Anal cytology	X		—		—		—		—		—		—		—		—	
	Hepatitis B rapid test	X		—		—		—		—		X^p^		X^p^		X^p^		X^e^	
	Hepatitis B serology	X^q^		—		—		—		—		X^q^		X^q^		X^q^		X^e^	
	Hepatitis C rapid test	X		—		—		—		—		X^r^		X^r^		X^r^		X^e^	
	Anti-HCV	X^s^		—		—		—		—		X^s^		X^s^		X^s^		X^e^	
	RNA Hepatitis C viral load	X^t^		—		—		—		—		X^t^		X^t^		X^t^		X^e^	
	Treponemal syphilis rapid test	X		—		X		X		X		X		X		X		X^e^	
	Non-treponemal syphilis testing (VDRL)	X^u^		—		X^u^		X^u^		X^u^		X^u^		X^u^		X^u^		X^e^	
	DBS^v^ (PrEP adherence assessment)	X^w^		X^w^		X^w^		X^w^		X^w^		X^w^		X^w^		X^w^		X^e^	
	Creatinine	X^x^		X^f^		X^f^		X^x^		X^f^		X^x^		X^x^		X^f^		X^e^	
	Complete blood count	X^f^		X^f^		X^f^		X^f^		X^f^		X^f^		X^f^		X^f^		X^f^	

^a^RDS: respondent-driven sampling.

^b^Withdrawn or discontinued participants before the final visit.

^c^Not applicable

^d^Only for HIV-negative participants in a prior visit.

^e^If necessary.

^f^Only for postexposure prophylaxis use.

^g^Only for HIV-positive participants or HIV-negative participants with recent HIV exposition according to INI-Fiocruz guidelines (HIV acute infection screening).

^h^Only for HIV-negative participants with recent HIV exposition according to INI-Fiocruz guidelines (HIV acute infection screening).

^i^Only to HIV-positive participants or to participants with HIV rapid test, HIV RNA Pool or HIV RNA viral load positive result.

^j^Only to participants with HIV rapid test, HIV RNA Pool or HIV RNA viral load positive result, with negative HIV rapid test in a prior visit.

^k^Only HIV-positive participants ART naïve

^l^Only HIV-positive participants with prior ART use (before study initiation).

^m^CT: *Chlamydia trachomatis.*

^n^NG: *Neisseria gonorrhea.*

^o^HPV: human papilloma virus.

^p^Only for negative Hepatitis B rapid test at baseline.

^q^Only for positive Hepatitis B rapid test.

^r^Only for negative Hepatitis C rapid test at baseline.

^s^Only for positive Hepatitis C rapid test.

^t^Only for positive anti-HCV.

^u^Only treponemal syphilis rapid test.

^v^DBS: Dried blood spot.

^w^Only for participants using PrEP.

^x^Only for participants using PrEP or PEP.

### Analysis Plan for the Technology-Based Adherence Intervention

We will measure exposure to the intervention, including the number of received and read WhatsApp messages during the 24-week intervention period. Intervention exposure and outcome data will be analyzed using multivariable logistic generalized estimating equations (GEE) with exchangeable correlation structures to account for repeated measures over time for each participant. GEE models will assess the association between intervention exposure measures (eg, receipt of intervention and intervention dosage) and primary outcomes related to the HIV care and PrEP continuum.

## Results

To date, the Conectad@s Project has made significant progress despite the still ongoing COVID-19 pandemic. During 2020, we prepared the questionnaires for the RDS survey, intervention, and formative phase. We also discussed the best approach to move forward with the study in the context of the COVID-19 pandemic. The formative stage of the study started in January 2021. We conducted 20 individual interviews from January 12 to February 4, 2021, and three focus group discussions on February 4, 10, and 24, 2021. The study team prepared a COVID-19 plan that includes assessing COVID-19 symptoms, screening by phone prior to study attendance onsite before study visit, as well as a strong recommendation of facemask use and social distancing. Planned activities are indicated in Table 3.

**Table 3:** Planned activities for the Conectad@s Project.

Activities	2022				2023				
	Q1	Q2	Q3	Q4	Q1		Q2	Q3	Q4
Formative phase analysis	x	—^a^	—	—	—		—	—	—
Dissemination of formative results	—	x	—	—	—		—	—	—
Manual of operations approval	x	—	—	—	—		—	—	—
Training	x	—	—	—	—		—	—	—
Investigators’ meetings	x	—	—	—	x		—	—	—
RDS^b^ survey and intervention enrollment	x	x	x	—	—		—	—	—
Follow-up for intervention	—	x	x	x	x		x	—	—
RDS survey analysis	—	—	x	—	—		—	—	—
Dissemination of RDS results	—	—	—	x	—		—	—	—
Intervention analysis	—	—	—	—	—		—	x	—
Dissemination of intervention results	—	—	—	—	—		—	—	x

^a^Not applicable.

^b^RDS: respondent-driven sampling.

## Discussion

The Conectad@s Project is a vanguard study that will apply for the first time an RDS-based survey for YMSM in Brazil integrated with a technology-based adherence intervention. The results of the RDS survey will allow us to estimate HIV prevalence and incidence using recency testing and to measure HIV biomarkers near the onset of risky behavior among Brazilian YMSM. The intervention study will contribute to developing intervention-based adherence strategies to support HIV care and prevention among a highly vulnerable population. Data will lay the groundwork to adapt and implement all strategies to identify the barriers to the earliest possible diagnosis, immediate ART initiation, PrEP uptake, and detecting new clusters of HIV transmission.

## Appendix

Multimedia Appendix 1 Peer-review report by the Center for Scientific Review Special Emphasis Panel - RFA-AI-18-054 U.S.-Brazil Collaborative Biomedical Research Program (National Institutes of Health, USA).

## Abbreviations

ART: antiretroviral therapy
GEE: generalized estimating equations
INI-Fiocruz: Instituto Nacional de Infectologia Evandro Chagas, Fundação Oswaldo Cruz
LGBTQIA+: lesbian, gay, bisexual, transgender, queer/questioning, intersex, and asexual/aromantic/agender
MSM: men who have sex with men
PrEP: preexposure prophylaxis
RDS: respondent-driven sampling
STI: sexually transmitted infections
SUS: Sistema Único de Saúde (Brazilian Public health System)
VL: HIV RNA viral load
YMSM: young men who have sex with men

## References

[1] Brazil, Ministry of Health Boletim Epidemiológico HIV/Aids 2020 Internet. 2020.

[2] Luz P, Veloso V, Grinsztejn B (2019). The HIV epidemic in Latin America: accomplishments and challenges on treatment and prevention. Curr Opin HIV AIDS.

[3] Araújo MAL, Montagner MA, da Silva RM, Lopes FL, de Freitas MM (2009). Symbolic violence experienced by men who have sex with men in the primary health service in Fortaleza, Ceará, Brazil: negotiating identity under stigma. AIDS Patient Care STDS.

[4] Sabidó M, Kerr LRFS, Mota RS, Benzaken AS, de A Pinho A, Guimaraes MDC, Dourado I, Merchan-Hamman E, Kendall C (2015). Sexual Violence Against Men Who Have Sex with Men in Brazil: A Respondent-Driven Sampling Survey. AIDS Behav.

[5] Kerr L, Kendall C, Guimarães MDC, Salani Mota R, Veras M, Dourado I, Maria de Brito A, Merchan-Hamann E, Pontes A, Leal A, Knauth D, Castro A, Macena R, Lima L, Oliveira L, Cavalcantee MDS, Benzaken A, Pereira G, Pimenta C, Pascom A, Bermudez X, Moreira R, Brígido LFM, Camillo A, McFarland W, Johnston L (2018). HIV prevalence among men who have sex with men in Brazil: results of the 2nd national survey using respondent-driven sampling. Medicine (Baltimore).

[6] Kerr LRFS, Mota RS, Kendall C, Pinho ADA, Mello MB, Guimarães MDC, Dourado I, de Brito AM, Benzaken A, McFarland W, Rutherford G, HIVMSM Surveillance Group (2013). HIV among MSM in a large middle-income country. AIDS.

[7] Guimarães MDC, Kendall C, Magno L, Rocha G, Knauth D, Leal A, Dourado I, Veras M, Brito AM, Kerr LRFS, Brazilian HIV/MSM Surveillance Group (2018). Comparing HIV risk-related behaviors between 2 RDS national samples of MSM in Brazil, 2009 and 2016. Medicine (Baltimore).

[8] Grant RM, Lama JR, Anderson PL, McMahan V, Liu AY, Vargas L, Goicochea P, Casapía M, Guanira-Carranza JV, Ramirez-Cardich ME, Montoya-Herrera O, Fernández T, Veloso VG, Buchbinder SP, Chariyalertsak S, Schechter M, Bekker L, Mayer KH, Kallás EG, Amico KR, Mulligan K, Bushman LR, Hance RJ, Ganoza C, Defechereux P, Postle B, Wang F, McConnell JJ, Zheng J, Lee J, Rooney JF, Jaffe HS, Martinez AI, Burns DN, Glidden DV, iPrEx Study Team (2010). Preexposure chemoprophylaxis for HIV prevention in men who have sex with men. N Engl J Med.

[9] Grinsztejn B, Hoagland B, Moreira RI, Kallas EG, Madruga JV, Goulart S, Leite IC, Freitas L, Martins LMS, Torres TS, Vasconcelos R, De Boni RB, Anderson PL, Liu A, Luz PM, Veloso VG, PrEP Brasil Study Team (2018). Retention, engagement, and adherence to pre-exposure prophylaxis for men who have sex with men and transgender women in PrEP Brasil: 48 week results of a demonstration study. Lancet HIV.

[10] Hoagland B, Moreira RI, De Boni RB, Kallas EG, Madruga JV, Vasconcelos R, Goulart S, Torres TS, Marins LMS, Anderson PL, Luz PM, Costa Leite ID, Liu AY, Veloso VG, Grinsztejn B, PrEP Brasil Study Team (2017). High pre-exposure prophylaxis uptake and early adherence among men who have sex with men and transgender women at risk for HIV Infection: the PrEP Brasil demonstration project. J Int AIDS Soc.

[11] Marins LMS, Torres TS, Leite IDC, Moreira RI, Luz PM, Hoagland B, Kallas EG, Madruga JV, Liu AY, Anderson PL, Grinsztejn B, Veloso VG (2019). Performance of HIV pre-exposure prophylaxis indirect adherence measures among men who have sex with men and transgender women: Results from the PrEP Brasil Study. PLoS One.

[12] Torres TS, Konda KA, Vega-Ramirez EH, Elorreaga OA, Diaz-Sosa D, Hoagland B, Diaz S, Pimenta C, Bennedeti M, Lopez-Gatell H, Robles-Garcia R, Grinsztejn B, Caceres C, Veloso VG, ImPrEP Study Group (2019). Factors Associated With Willingness to Use Pre-Exposure Prophylaxis in Brazil, Mexico, and Peru: Web-Based Survey Among Men Who Have Sex With Men. JMIR Public Health Surveill.

[13] Torres TS, Luz PM, De Boni RB, de Vasconcellos MT, Hoagland B, Garner A, Moreira RI, Veloso VG, Grinsztejn B (2019). Factors associated with PrEP awareness according to age and willingness to use HIV prevention technologies: the 2017 online survey among MSM in Brazil. AIDS Care.

[14] Luz PM, Torres TS, Almeida-Brasil CC, Marins LMS, Veloso VG, Grinsztejn B, Cox J, Moodie EEM (2021). High-Risk Sexual Behavior, Binge Drinking and Use of Stimulants are Key Experiences on the Pathway to High Perceived HIV Risk Among Men Who Have Sex with Men in Brazil. AIDS Behav.

[15] Torres TS, Marins LMS, Veloso VG, Grinsztejn B, Luz PM (2019). How heterogeneous are MSM from Brazilian cities? An analysis of sexual behavior and perceived risk and a description of trends in awareness and willingness to use pre-exposure prophylaxis. BMC Infect Dis.

[16] Pascom A, Meireles M, Benzaken A (2018). Sociodemographic determinants of attrition in the HIV continuum of care in Brazil, in 2016. Medicine (Baltimore).

[17] Macinko J, Mullachery P, Proietti FA, Lima-Costa MF (2012). Who experiences discrimination in Brazil? Evidence from a large metropolitan region. Int J Equity Health.

[18] Fitzgerald-Husek A, Van Wert MJ, Ewing WF, Grosso AL, Holland CE, Katterl R, Rosman L, Agarwal A, Baral SD (2017). Measuring stigma affecting sex workers (SW) and men who have sex with men (MSM): A systematic review. PLoS One.

[19] de Castro CAV, Grinsztejn B, Veloso VG, Bastos FI, Pilotto JH, Morgado MG (2010). Prevalence, estimated HIV-1 incidence and viral diversity among people seeking voluntary counseling and testing services in Rio de Janeiro, Brazil. BMC Infect Dis.

[20] Coelho LE, Torres TS, Veloso VG, Grinsztejn B, Jalil EM, Wilson EC, McFarland W (2021). The Prevalence of HIV Among Men Who Have Sex With Men (MSM) and Young MSM in Latin America and the Caribbean: A Systematic Review. AIDS Behav.

[21] Arayasirikul S, Chen Y, Jin H, Wilson E (2016). A Web 2.0 and Epidemiology Mash-Up: Using Respondent-Driven Sampling in Combination with Social Network Site Recruitment to Reach Young Transwomen. AIDS Behav.

[22] Gile KJ, Handcock MS (2010). Respondent-Driven Sampling: An Assessment of Current Methodology. Sociol Methodol.

[23] Sanchez MA, Scheer S, Shallow S, Pipkin S, Huang S (2014). Epidemiology of the viral hepatitis-HIV syndemic in San Francisco: a collaborative surveillance approach. Public Health Rep.

[24] Abdul-Quader AS, Heckathorn DD, Sabin K, Saidel T (2006). Implementation and analysis of respondent driven sampling: lessons learned from the field. J Urban Health.

[25] Arayasirikul S, Cai X, Wilson EC (2015). A Qualitative Examination of Respondent-Driven Sampling (RDS) Peer Referral Challenges Among Young Transwomen in the San Francisco Bay Area. JMIR Public Health Surveill.

[26] Johnston LG, Chen Y, Silva-Santisteban A, Raymond HF (2013). An empirical examination of respondent driven sampling design effects among HIV risk groups from studies conducted around the world. AIDS Behav.

[27] Truong HM, Grasso M, Chen Y, Kellogg TA, Robertson T, Curotto A, Steward WT, McFarland W (2013). Balancing theory and practice in respondent-driven sampling: a case study of innovations developed to overcome recruitment challenges. PLoS One.

[28] Grebe E, Welte A, Johnson LF, van Cutsem G, Puren A, Ellman T, Etard J, Huerga Helena, Consortium for the EvaluationPerformance of HIV Incidence Assays (CEPHIA) (2018). Population-level HIV incidence estimates using a combination of synthetic cohort and recency biomarker approaches in KwaZulu-Natal, South Africa. PLoS One.

[29] Brazil, Ministry of Health Protocolo Clínico e Diretrizes Terapêuticas para Atenção Integral às Pessoas com Infecções Sexualmente Transmissíveis (IST) Internet. 2020.

[30] Ministry OH, Brazil Protocolo Clínico e Diretrizes Terapêuticas para Hepatite C e Coinfecções Internet. 2020.

[31] Bohl DD, Raymond HF, Arnold M, McFarland W (2009). Concurrent sexual partnerships and racial disparities in HIV infection among men who have sex with men. Sex Transm Infect.

[32] Pinkerton SD, Galletly CL, McAuliffe TL, DiFranceisco W, Raymond HF, Chesson HW (2010). Aggregate versus individual-level sexual behavior assessment: how much detail is needed to accurately estimate HIV/STI risk?. Eval Rev.

[33] Costa JDM, Torres TS, Coelho LE, Luz PM (2018). Adherence to antiretroviral therapy for HIV/AIDS in Latin America and the Caribbean: Systematic review and meta-analysis. J Int AIDS Soc.

[34] Vale FC, Santa-Helena ETD, Santos MA, Carvalho WMDES, Menezes PR, Basso CR, Silva MH, Alves AM, Nemes MIB (2018). Development and validation of the WebAd-Q Questionnaire to monitor adherence to HIV therapy. Rev Saude Publica.

[35] Almeida-Brasil CC, Nascimento ED, Silveira MR, Bonolo PDF, Ceccato MDGB (2019). New patient-reported outcome measure to assess perceived barriers to antiretroviral therapy adherence: the PEDIA scale. Cad Saude Publica.

[36] Luz PM, Torres TS, Almeida-Brasil CC, Marins LMS, Bezerra DRB, Veloso VG, Grinsztejn B, Harel D, Thombs BD (2020). Translation and validation of the Short HIV Stigma scale in Brazilian Portuguese. Health Qual Life Outcomes.

[37] Torres TS, Bastos LS, Kamel L, Bezerra DR, Fernandes NM, Moreira RI, Garner A, Veloso VG, Grinsztejn B, De Boni RB (2020). Do men who have sex with men who report alcohol and illicit drug use before/during sex (chemsex) present moderate/high risk for substance use disorders?. Drug Alcohol Depend.

[38] Torres TS, De Boni RB, de Vasconcellos MT, Luz PM, Hoagland B, Moreira RI, Veloso VG, Grinsztejn B (2018). Awareness of Prevention Strategies and Willingness to Use Preexposure Prophylaxis in Brazilian Men Who Have Sex With Men Using Apps for Sexual Encounters: Online Cross-Sectional Study. JMIR Public Health Surveill.

[39] Coelho LE, Torres TS, Veloso VG, Landovitz RJ, Grinsztejn B (2019). Pre-exposure prophylaxis 2.0: new drugs and technologies in the pipeline. Lancet HIV.

[40] Torres TS, Luz PM, Marins LMS, Bezerra DRB, Almeida-Brasil CC, Veloso VG, Grinsztejn B, Harel D, Thombs BD (2021). Cross-cultural adaptation of the Perceived Risk of HIV Scale in Brazilian Portuguese. Health Qual Life Outcomes.

[41] Blair KJ, Torres TS, Hoagland B, Bezerra DRB, Veloso VG, Grinsztejn B, Clark J, Luz PM (2022). Pre-exposure prophylaxis use, HIV knowledge, and internalized homonegativity among men who have sex with men in Brazil: A cross-sectional study. The Lancet Regional Health - Americas.

[42] Torres TS, Cox J, Marins LM, Bezerra DR, Veloso VG, Grinsztejn B, Luz PM (2020). A call to improve understanding of Undetectable equals Untransmittable (U = U) in Brazil: a web-based survey. J Int AIDS Soc.

[43] Moreno AL, DeSousa DA, Souza AMFLP, Manfro GG, Salum GA, Koller SH, Osório FL, Crippa JAS (2016). Factor Structure, Reliability, and Item Parameters of the Brazilian-Portuguese Version of the GAD-7 Questionnaire. Temas Psicol.

[44] Santos IS, Tavares BF, Munhoz TN, Almeida LSPD, Silva NTBD, Tams BD, Patella AM, Matijasevich A (2013). Sensibilidade e especificidade do Patient Health Questionnaire-9 (PHQ-9) entre adultos da população geral. Cad Saúde Pública.

[45] Osório FL,  Da Silva TDA, Dos Santos RG, Chagas MHN, Chagas NMS, Sanches RF, Crippa JADS (2017). Posttraumatic Stress Disorder Checklist for DSM-5 (PCL-5): transcultural adaptation of the Brazilian version. Arch Clin Psychiatry (São Paulo).

[46] Scandurra C, Amodeo AL, Valerio P, Bochicchio V, Frost DM (2017). Minority Stress, Resilience, and Mental Health: A Study of Italian Transgender People. Journal of Social Issues.

[47] Bastos JL, Faerstein E, Celeste RK, Barros AJD (2012). Explicit discrimination and health: development and psychometric properties of an assessment instrument. Rev Saude Publica.

[48] Torres TS, Luz PM, Bezerra DRB, Almeida-Brasil CC, Marins LMS, Veloso VG, Grinsztejn B, Harel D, Thombs BD (2021). Translation and validation in Brazilian Portuguese of the reactions to homosexuality scale. Psychol Health Med.

[49] Scanavino MDT, Ventuneac A, Rendina HJ, Abdo CHN, Tavares H, Amaral MLSD, Messina B, Reis SCD, Martins JPLB, Gordon MC, Vieira JC, Parsons JT (2016). Sexual Compulsivity Scale, Compulsive Sexual Behavior Inventory, and Hypersexual Disorder Screening Inventory: Translation, Adaptation, and Validation for Use in Brazil. Arch Sex Behav.

[50] Torres TS, Hoagland B, Bezerra DRB, Garner A, Jalil EM, Coelho LE, Benedetti M, Pimenta C, Grinsztejn B, Veloso VG (2021). Impact of COVID-19 Pandemic on Sexual Minority Populations in Brazil: An Analysis of Social/Racial Disparities in Maintaining Social Distancing and a Description of Sexual Behavior. AIDS Behav.

[51] Segall-Corrêa AM, Marin-Leon L (2015). A segurança alimentar no Brasil: proposição e usos da escala brasileira de medida da insegurança alimentar (EBIA) de 2003 a 2009. Segur Aliment Nutr.

[52] Brazil, Ministry of Health (2018). Protocolo Clínico e Diretrizes Terapêuticas para Profilaxia Pré-Exposição (PrEP) de Risco à Infecção pelo HIV Internet.

