# Supplementary material for: A Technology-Based Intervention Among Young Men Who Have Sex With Men and Nonbinary People (The Conectad@s Project): Protocol for A Vanguard Mixed Methods Study
Source: JMIR Res Protoc. 2022 Jan 13;11(1):e34885. doi: 10.2196/34885 (PMC8796043; doi:10.2196/34885)
Supplement: Multimedia Appendix 1 [file resprot_v11i1e34885_app1.pdf]

SUMMARY STATEMENT
(Privileged Communication)

PROGRAM CONTACT:
Cherlynn Mathias
240-292-4791
cmathias@niaid.nih.gov

Release Date: 07/26/2019
Revised Date:

Application Number: 1 R01 AI149627-01

Principal Investigators (Listed Alphabetically):

GRINSZTEJN, BEATRIZ
MCFARLAND, WILLIAM (Contact)

Applicant Organization: PUBLIC HEALTH FOUNDATION ENTERPRISES

Review Group: ZRG1 IMM-S (50)
Center for Scientific Review Special Emphasis Panel
RFA-AI-18-054 U.S.-Brazil Collaborative Biomedical Research Program
AIDS - EXP. REV.

Meeting Date: 07/18/2019
Council: OCT 2019
Requested Start: 12/01/2019
RFA/PA: AI18-054
PCC: A22

Project Title: San Francisco-Rio de Janeiro collaborative research on HIV prevalence, risk, and biomedical prevention interventions among young men who have sex with men
SRG Action: Impact Score:20
Next Steps: Visit https://grants.nih.gov/grants/next_steps.htm
Human Subjects: 30-Human subjects involved - Certified, no SRG concerns
Animal Subjects: 10-No live vertebrate animals involved for competing appl.
Gender: 1A-Both genders, scientifically acceptable
Minority: 1A-Minorities and non-minorities, scientifically acceptable
Age: 1A-Children, Adults, Older Adults, scientifically acceptable

Project Year	Direct Costs Requested	Estimated Total Cost
1	100,000	110,006
2	100,000	110,006
3	100,000	110,006
4	100,000	110,006
TOTAL	400,000	440,024

1R01AI149627-01 MCFARLAND, WILLIAM

RESUME AND SUMMARY OF DISCUSSION: This R01 application will attempt to address the HIV epidemic among young men in Brazil. Three complementary aims are proposed that include 1) a cross-sectional survey of MSM, ages 18-24, to determine HIV prevalence and onset risk behavior; 2) development of a technology-based intervention for rapid ART and PrEP engagement and retention in this population; 3) qualitative study of age 15-17 MSM to better understand onset risk in this population and increase participation in HIV prevention and intervention programs. The research team is outstanding with a history of demonstrated integrative and collaborative efforts between the U.S. and Brazil. Prior co-investigative publications support a strong rigor of the prior research and provide strength for significance and approach. Clearly defined responsibilities and division of work for the proposed studies are provided. Committee members were highly enthusiastic about Aims 1 and 2 with rigorous study designs that build upon previous experience and success. Potential pitfalls were addressed with well-reasoned alternative solutions. However, feasibility of Aim 3 was less certain as the Aim was considered to be underdeveloped, ambitious and rather inspirational. Overall, weaknesses were overshadowed by the many strengths with Committee members giving very strong enthusiasm as the collective U.S. and Brazil efforts have potential to tackle and reduce a highly critical public health concern in Brazil.

DESCRIPTION (provided by applicant): As San Francisco's HIV epidemic is set to reach zero infections, Rio de Janeiro is experiencing a resurgence in new reported HIV cases among MSM. San Francisco's success can be attributed to effective biomedical interventions, namely: rapid diagnosis, immediate ART, sustained viral load suppression, wide uptake of PrEP, and interrupting new clusters of transmission. In Brazil, however, uptake of these biomedical interventions has so far been insufficient to reverse the epidemic, despite being the first low/middle income country to provide free ART to all persons with HIV, participation in clinical trials proving PrEP efficacy, and national policy to provide free PrEP. Meanwhile, young MSM, particularly Black/Afro-Brazilians, have high HIV incidence, low viral suppression, and poor PrEP adherence. We therefore propose collaborative research between the San Francisco Department of Public Health and the Oswaldo Cruz Foundation (Fiocruz) in Rio de Janeiro. We will focus on identifying the drivers of HIV infection, ways to address barriers to biomedical interventions, and identifying the hurdles to including young MSM in biomedical research. Methods include a cross-sectional survey of MSM age 18-24 using respondent-driven sampling adapted for social media-based sampling; development of a technology-based rapid ART and PrEP engagement and retention intervention; and a qualitative study of MSM age 15-17, their parents, and providers of sexual health services to better understand the onset of risk and the feasibility of increasing inclusion of minors in biomedical HIV research and intervention programs. San Francisco stands as an American test case for the elimination of HIV, yet remaining challenges exist in reaching young and minority MSM. Rio de Janeiro is an engine of HIV research in South America, yet faces challenges in reaping the benefit of proven biomedical interventions for MSM. The proposed US-Brazil research collaboration will provide mutual benefit in increasing the effectiveness of biomedical interventions to reverse the HIV epidemic among MSM in Brazil and address remaining disparities in the US.

PUBLIC HEALTH RELEVANCE: We propose to start a line of research to help turn the tide on the HIV epidemic among young men who have sex with men (MSM) in Brazil. Researchers from the San Francisco health department will team up with scientists in Rio de Janeiro. Three joint projects are proposed: a survey of young MSM to measure the onset of risk for HIV, the development of a social media application to link young men to HIV care and PrEP, and an investigation of the concerns of parents, doctors, and young MSM about including minors in biomedical HIV programs and research.

CRITIQUE 1:

Significance: 2
Investigator(s): 1
Innovation: 2
Approach: 2
Environment: 1

Overall Impact: This is a joint research proposal between the San Francisco Department of Public Health and the Oswald Cruz Foundation (Fiocruz) in Rio de Janeiro addressing the urgent public health problem in Brazil of growing HIV prevalence among young men having sex with men (MSM). The proposed project will use respondent-driven sampling (RSD) and social media, the methods that contributed to decline of HIV transmission in SF, to 1) determine HIV prevalence, onset of risk behavior, and barriers to intervention among young adult MSM in Rio de Janeiro; 2) implement rapid PrEP, ART, and patient retention in this population; and 3) attempt to increase participation of adolescent MSM in HIV prevention research and programs. The project builds on the recent successful collaboration between the two groups that started in 2015 and determined HIV prevalence in transgender women in Rio de Janeiro. The importance and rigor of this research is indicated by joint publications in top biomedical journals. The present project leverages the well-established clinical HIV research infrastructure at INI-Fiocruz, the experience of the Fiocruz team with the RDS methodology gained in the transwomen study, and the experience of the SF team in RDS, clinical HIV research, interventions, and biomarker analysis. Owing in part to this unique combination of expertise and in part to the large MSM population at risk, the proposed research partnership has high chances of success, especially in the first two aims of the project. The feasibility of Aim 3 is more difficult to assess and studies in this Aims will not start before year 3 of the project. However, targeting this subpopulation for research is both novel and potentially critical for understanding the root causes of the growing HIV epidemic among MSM in Brazil. Reciprocal benefits in helping to address the remaining gender and race disparities in HIV treatment in the US are expected. Overall, this is an outstanding well-integrated collaborative project which addresses a key public health problem in Brazil and takes full advantage of the complementary research capacities of the partnering US and Brazilian teams.

1. Significance:

Strengths

- The proposed project extends the already proven and productive research partnership between the SF and Rio de Janeiro groups.
- Joint publications in top biomedical journals attest to the importance and scientific rigor of past collaborative studies.
- The proposed project will further stimulate knowledge and technology transfer between the US and Brazil, an important consideration given the US team contribution to arresting HIV spread in San Francisco and the alarming resurgence of HIV infection in Rio de Janeiro.
- The focus of the current program on young MSM reflects the urgent public health problem in Brazil caused by steep rise in HIV infections among MSM since 2012, despite free access to ART.
- The experience of the SF group in social networks-based methods (e.g., RDS) for patient recruitment and intervention could be key to control of HIV epidemic in Brazil, especially if the approach can be applied to targeting adolescent MSM at risk for HIV, as suggested in the application.
- The project may generate reciprocal benefits for addressing the remaining gender and race disparities in HIV treatment in the US.

Weaknesses

- The proposed inclusion of adolescent MSM in HIV research and interventions will not be ready for implementation before year 3 of the project.

2. Investigator(s):

Strengths

- The PI, Dr. Willi McFarland, is the Director of the Center for Public Health Research within SFDPH and Professor of Medicine at UCSF. Dr. McFarland is a renowned expert in HIV epidemiology and prevention research including application of RDS methodology for patient recruitment and interventions, has over 400 publications, and managed or served as a co-investigator in over 100 research projects in the US and abroad. Dr. McFarland has extensive experience in directing complex multidisciplinary research projects and has been involved in HIV research in Brazil for many years, including directing HIV studies in Brazil, interacting with local research and health administrations, and training Brazilian scientists. He is exquisitely qualified to serve as a leading PI on the present study.
- The MPI, Dr. Beatriz Grinsztejn, is the Head of the STD/AIDS Clinical Research Laboratory at the National Institute of Infectious Diseases (INI) at the Oswaldo Cruz Foundation-FIOCRUZ and the Director of the Fiocruz HIV/AIDS Clinical Trials Unit. Dr. Grinsztejn is the leading HIV/AIDS physician-scientist in Brazil with 30 years of experience, proven leadership in managing clinical trials for the disease, international reputation, over 290 publications, and long track record of research funding. She is extremely well qualified to direct the Brazil component of the proposed project.
- Other members of the research partnership were well chosen for their essential contributions to different aspects of the project.

Weaknesses

- None noted.

3. Innovation:

Strengths

- Sophisticated biomarker approach to identify acute and recent infections and clusters of transmission in MSM
- Plan to develop Brazil-customized, technology-based intervention platform for PrEP and ART adherence.
- Plan to include MSM minors in future biomedical HIV research in Brazil; if successfully implemented, the approach developed in Brazil may help in recruiting similar difficult to reach risk subgroups in the US.

Weaknesses

- The digital/social media-based sampling methodology in adult populations at risk for HIV is not new at Fiocruz; application to young MSM is novel.
- The HIV diagnoses among Black/African American MSM in SF increased between 2016 and 2018, despite the overall success of RDS methods in the city. It is thus not sure that the same outreach methodology will be effective in slowing HIV spread among Black/Afro-Brazilians.

4. Approach:

Strengths

- Very well and clearly written, well integrated application, fully responsive to the goals of the RFA.

- The US and Brazil research teams partnering in this project have proven track record of productive collaboration, suggesting minimal preparatory steps before project is started.
- The responsibilities of each partner/research site are clearly described, including the research expertise and contributions of individual co-investigators, flow of travel and work between the two sites, integration of research efforts, communication plans, scientific direction decision making, and conflicts resolution.
- Aims 1 and 2 are the strongest in the application and their detailed design, both in the clinical and analytical parts, indicates synergy between the two partners in the thought process and research integration. There are detailed considerations for achieving statistical power of analysis, contingency plans for delays or difficulties in patient accrual or retentions, plans for adapting RDS to local circumstances, etc.
- While ambitious, aim 1 and 2 will likely produce interpretable results, although the rate of success in the proposed interventions in Aim 2 may not be fully known before the end of the project.
- Accomplishment of Aim 1 and 2 and preparatory work toward Aim 3 will qualify as an unalloyed success of the project.

Weaknesses

- Aim 3 is highly innovative and likely important for understanding the dynamics of HIV epidemic among young MSM in Brazil. However, it requires significant preparatory work. The investigators are aware of this and they do not expect IRB approval before mid-year 3 of the project, leaving limited time for recruitment and research. Given these constraints, this Aim is deemed inspirational rather than practical.

5. Environment:

Strengths

- Superb at both sites.

Weaknesses

- None noted.

Protections for Human Subjects:

Acceptable Risks and/or Adequate Protections

- IRB pending

Inclusion Plans:

- Sex/Gender: Distribution justified scientifically.
- Race/Ethnicity: Distribution justified scientifically.
- Inclusion/Exclusion Based on Age: Distribution justified scientifically.
- Inclusion plans reflect the MSM nature of the project and ethnic realities in Brazil.

Vertebrate Animals:

Not Applicable (No Vertebrate Animals)

Biohazards:

Not Applicable (No Biohazards)

Applications from Foreign Organizations:
Justified

- As described in the RFA.

Resource Sharing Plans:
Acceptable

- Well described.

Authentication of Key Biological and/or Chemical Resources:
Acceptable

- (described in text, not as a separate section)

Budget and Period of Support:
Recommend as Requested

CRITIQUE 2:

Significance: 1
Investigator(s): 2
Innovation: 1
Approach: 3
Environment: 2

Overall Impact: The study proposes collaborative research between the San Francisco Department of Health and the Oswaldo Cruz Foundation in Rio de Janeiro. The team proposed three sequential studies in three separate study aims with different but interrelated purposes. The first is a cross-sectional study of prevalence and incidence of HIV infection in young MSM, ages 18-24, using biomarkers of recent and acute infection, together with a comprehensive survey of onset of sexual behavior, risk behavior and barriers to uptake of PrEP and TasP. The second study aims to develop and then test a technology based intervention using WhatsApp to promote ART and PrEP adherence using reminders and other information. The third study is a qualitative study of young MSM ages 15-17 to identify barriers and potential points of intervention for uptake of ART and PrEP. Overall, specification of aim 1 is stronger than the other two aims and most of the enthusiasm is for this aim, with primary concerns for Aims 2-3. The investigative teams are very strong, the potential for building collaboration is well considered, and the study has promise for public health impact.

1. Significance:

Strengths

- The proposal of three related studies is robust for generating opportunities for collaboration.
- The focus on very young (15-17) and young (18-24) Afro/Black MSM in all aims is well justified and presents a strong potential for informing intervention development.
- The focus on ART and PrEP uptake and adherence among YMSM promises to address a gap in knowledge in the Brazilian context.

- A strong history of collaboration between the investigators for work internationally with MSM strengthens the proposal.

Weaknesses

- A discussion of major environmental and structural differences that contribute to success in SF, but may not translate to Rio (population size, density, political will/support, etc) would strengthen the proposal.

2. Investigator(s):

Strengths

- The investigators from both sites are well qualified to carry out this research with expertise in the sampling approach, qualitative and quantitative methods, and intervention development.
- There is a strong history of collaboration between the partners with evidence of expertise in HIV prevention in Brazil among MSM and other locations internationally.

Weaknesses

- The minimal Portuguese language capacity on the US team, given they will lead qualitative analysis, limits interpretation and impact. None of the Brazilian investigators are identified as participating in the qualitative analysis.
- None of the investigators have particular expertise regarding the barriers to uptake and maintenance in care among adolescent MSM.

3. Innovation:

Strengths

- The inclusion of biomarkers of recent and acute infection among young MSM in the context of recent sexual initiation is innovative.
- The study of barriers to biomedical prevention approaches among very young MSM, ages 15-17 is innovative.

Weaknesses

- Use of electronic means to distribute RDS coupons is now common practice and thus not particularly innovative.

4. Approach:

Strengths

- The application approach is clearly collaborative and will draw on strengths of both the Brazilian and US teams.
- The use of biomarkers of recent and acute HIV infection, as well as assessment of common STIs is a strength.
- The application includes well-developed methods and measures, building on prior research with MSM in the US and other international locations is a strength.
- The overall concept in linking the three studies, observational, pilot intervention and qualitative to inform future intervention development is strong.

Weaknesses

- For Aim 1, the investigators don't specify the metrics collected to inform assessment of RDS assumptions, particularly the size and segregation of networks that would lead to the problems identified in the narrative (e.g., bottlenecks, seed bias).

- For Aim 1, detection of clusters of HIV infection may be limited both by the estimated prevalence of 10% (n=40) and targeting of social network members in the sampling approach (via RDS), rather than sexual network members.
- For Aim 2, the description of the WhatsApp intervention content, in general, is underdeveloped. General references are to weekly reminders (why weekly?) and factoid message option (no rationale for why this might promote adherence) are not well supported.
- For Aim 2, the research design is underdeveloped. It's not clear why with a relatively large sample, the investigators chose a pre-post design.
- For Aim 2, there does not appear to be any plans for a usability study to optimize the functionality of the intervention delivery (timing, frequency, dosage) before launching among the large sample.
- For Aim 2, the plan to assess intervention dosage via return reply may not be a reliable, i.e., potential for low response rate; may receive message, but not reply, and may diminish over time.
- For Aim 2, it's not clear how the primary outcome will be evaluated – in each group separately (HIV+/-) or collapsed together? How will PrEP adherence be measured? By self-report or biomarker? Will visit data be abstracted from medical records?
- For Aim 2, although the use of WhatsApp and SKEDit for this technology-based intervention is intuitive, the background doesn't include any data on use of WhatsApp in Brazil or feasibility of WhatsApp or SKEDit for the proposed intervention. Although described in the application as SMS, WhatsApp does not use SMS. It relies on downloading of the app and internet connection, thus describing the distribution of both of these (smartphone use, internet access) among the target population is important as part of feasibility.
- For Aim 3, there's no data to support feasibility of recruitment of parents of young MSM. Parents would need to be aware of their youth's status and willing to participate. How "out" are youth? Are parents likely to know about MSM status?
- For Aim 3, it's not clear whether or not the youth and parents are recruited as a dyad? This would have important implications for analyzing the data.
- There's no development phase or usability study listed in the timeline for the Aim 2 WhatsApp intervention.
- If the IRB approval for research with minors and waiving parent consent under Aim 3 is expected to be difficult, it may be prudent to allow more than 6 months in YR3 for preparation, submission and approval.

5. Environment:

Strengths

- The resources and facilities at FIOCRUZ, including lab capacity, are well described and justified.

Weaknesses

- There's no description of environment at Heluna Health as the awardee and fiscal agent. It's not clear what infrastructure supports are in place for international research, for example (i.e., for visa processing, international contracts negotiation, processing of invoices, banking issues, etc). Limited information about role of Heluna as fiscal agent is embedded in the budget justification and therefore difficult to evaluate.

Protections for Human Subjects:

Acceptable Risks and/or Adequate Protections

- There's no discussion of privacy concerns for use of mobile phones for intervention or use of 3rd party provider (WhatsApp, SKEDit) for hosting of messages, this might be taken for granted, but should be described to make it clear that the investigators understand related risks and related them in the consent form.
- The investigators propose to use internal monitoring procedures. Risk is minimal, but procedures for monitoring should be spelled out in a bit more detail.

Inclusion Plans:

- Sex/Gender: Distribution justified scientifically.
- Race/Ethnicity: Distribution justified scientifically.
- Inclusion/Exclusion Based on Age: Distribution justified scientifically.

Vertebrate Animals:

Not Applicable (No Vertebrate Animals)

Biohazards:

Not Applicable (No Biohazards)

Applications from Foreign Organizations:

Justified

- The investigators have justified the foreign component per the FOA.

Select Agents:

Not Applicable (No Select Agents)

Resource Sharing Plans:

Acceptable

Authentication of Key Biological and/or Chemical Resources:

Not Applicable (No Relevant Resources)

Budget and Period of Support:

Recommended budget modifications or possible overlap identified:

- The team should be a bit more explicit to explain how this project will be accomplished in the international site without any salary support for investigators.

CRITIQUE 3:

Significance: 1
Investigator(s): 1
Innovation: 1

Approach: 2
Environment: 1

Overall Impact: As highlighted in the application, San Francisco is striving to become one of the first cities in the United States to adequately control the HIV epidemic especially among MSM. The rising HIV epidemic among MSM youth in Rio de Janeiro is concerning and represents a public health emergency. The significance of this problem is duly noted. This application proposes to implement a multipronged approach to address drivers of the HIV infection among MSM youth in Rio de Janeiro. The proposal is highly innovative and builds on work that has already been accomplished in San Francisco adding to its potential success. The investigators have extensive experience with the proposed sampling methods as well as relevant expertise in clinical research in HIV prevention with Prep and ART. The collaboration between investigators in San Francisco and INI-FIOCRUZ provides a unique environment in which to study the proposed multipronged approach and represents a true partnership. The population based survey using novel respondent driven sampling; biomarkers to detect early HIV infection; technology based intervention to support ART and PREP adherence, and a qualitative study on ethical challenges for participation is well described and seems feasible. The proposal also takes advantage of free ART that is currently being provided by the Brazilian government. The authors adequately address setbacks and delays and provide alternative methods. Overall, this is an excellent application that addresses a highly significant problem in a novel and innovative way. The investigators are highly experienced, and the outcomes of this study have the potential to impact populations of MSM youth in Rio de Janeiro.

1. Significance:

Strengths

- This grant addresses a significant problem which is HIV transmission among youth MSM in Rio. 18% of MSM in Rio are infected with HIV and the majority are youth and Black or Afro/Brazilian. Preliminary data from co-I indicates very high risk behavior among young men age 18-24
- The combined expertise of the Brazilian and US based researchers addresses a key public health issue that would not otherwise be explored and provides a unique opportunity to reduce incident HIV infections among MSM youth in Rio

Weaknesses

- None noted.

2. Investigator(s):

Strengths

- Proposal is written as a true collaboration between American and Brazilian investigators
- Both PIs and co investigators have extensive experience with HIV prevention and are fully capable to carry out this research. The addition of investigators who work at the intersection of race, sexuality, and gender on HIV prevention adds tremendously to the proposal
- Dr. McFarland has collaborated with researchers in Brazil for over 17 years and has mentored 18 Brazilian scientists. Dr. Grinsztejn, the co PI, is the head of the NIH funded CTU in Rio and has mentored 25 young Brazilian scientists.

Weaknesses

- None noted.

3. Innovation:

Strengths

- Proposal is very innovative and builds on prior successful work in San Francisco. While San Francisco has seen an almost 60% drop in new HIV infections among MSM, the opposite is observed in Rio.
- Uses methods that have not been used in Brazil before such as respondent driven sampling and biomarkers for acute infection to identify patients who need ART and HIV negative youth who would qualify for PREP
- Takes advantage of strong infrastructure in Brazil with free ART and free PREP
- HIV phylogenetic testing is innovative

Weaknesses

- The multipronged approach for HIV prevention in San Francisco seems to have been less successful among Black MSM where HIV incidence has risen. The authors acknowledge this weakness and have contingency plans. The semi-structured interviews may also shed light on this problem.

4. Approach:

Strengths

- The proposal leverages experience in San Francisco to address a major problem in Rio.
- The application clearly states which partner of the collaboration will be responsible for each component of the specific aims.
- The application provides appropriate plans for collaborative research and adequately addresses delays and setbacks.
- The net effect on outcomes from the research will definitely result in synergy than if the projects were performed separately.
- The focus groups prior to development of recruitment methods should provide critical information to maximize sampling and understanding cultural and social barriers to recruitment.

Weaknesses

- The study is recognizably a pilot. Biomarkers for adherence to prep as well as incident infection would also be worth examining in the HIV negative cohort.

5. Environment:

Strengths

- Environment is highly appropriate for this proposal given the high incidence of HIV among MSM youth in Rio.
- Unique environment but lessons learned from this proposal would likely be significant in other LMICs with high HIV incidence among MSM youth.

Weaknesses

- None noted.

Protections for Human Subjects:

Acceptable Risks and/or Adequate Protections

Inclusion Plans:

- Sex/Gender: Distribution justified scientifically.
- Race/Ethnicity: Distribution justified scientifically.

- Inclusion/Exclusion Based on Age: Distribution justified scientifically.

Vertebrate Animals:
Not Applicable (No Vertebrate Animals)

Biohazards:
Acceptable

Applications from Foreign Organizations:
Justified

Resource Sharing Plans:
Acceptable

Authentication of Key Biological and/or Chemical Resources:
Not Applicable (No Relevant Resources)

Budget and Period of Support:
Recommend as Requested

THE FOLLOWING SECTIONS WERE PREPARED BY THE SCIENTIFIC REVIEW OFFICER TO SUMMARIZE THE OUTCOME OF DISCUSSIONS OF THE REVIEW COMMITTEE, OR REVIEWERS' WRITTEN CRITIQUES, ON THE FOLLOWING ISSUES:

PROTECTION OF HUMAN SUBJECTS: ACCEPTABLE

INCLUSION OF WOMEN PLAN: ACCEPTABLE

INCLUSION OF MINORITIES PLAN: ACCEPTABLE

INCLUSION ACROSS THE LIFESPAN PLAN: ACCEPTABLE

COMMITTEE BUDGET RECOMMENDATIONS: The budget was recommended as requested.

Footnotes for 1 R01 AI149627-01; PI Name: McFarland, William

NIH has modified its policy regarding the receipt of resubmissions (amended applications). See Guide Notice NOT-OD-14-074 at http://grants.nih.gov/grants/guide/notice-files/NOT-OD-14-074.html. The impact/priority score is calculated after discussion of an application by averaging the overall scores (1-9) given by all voting reviewers on the committee and multiplying by 10. The criterion scores are submitted prior to the meeting by the individual reviewers assigned to an application, and are not discussed specifically at the review meeting or calculated into the overall impact score. Some applications also receive a percentile

ranking. For details on the review process, see http://grants.nih.gov/grants/peer_review_process.htm#scoring.

MEETING ROSTER

Center for Scientific Review Special Emphasis Panel
CENTER FOR SCIENTIFIC REVIEW
RFA-AI-18-054 U.S.-Brazil Collaborative Biomedical Research Program

ZRG1 IMM-S (50)
07/18/2019

Notice of NIH Policy to All Applicants: Meeting rosters are provided for information purposes only. Applicant investigators and institutional officials must not communicate directly with study section members about an application before or after the review. Failure to observe this policy will create a serious breach of integrity in the peer review process, and may lead to actions outlined in NOT-OD-14-073 at https://grants.nih.gov/grants/guide/notice-files/NOT-OD-14-073.html and NOT-OD-15-106 at https://grants.nih.gov/grants/guide/notice-files/NOT-OD-15-106.html, including removal of the application from immediate review.

CHAIRPERSON(S)

HUGUENARD, JOHN R, PHD
PROFESSOR
DEPARTMENT OF NEUROLOGY AND NEUROLOGICAL SCIENCES
SCHOOL OF MEDICINE
STANFORD UNIVERSITY
STANFORD, CA 94304

MEMBERS

BERTRAM, EDWARD H, MD
PROFESSOR
DEPARTMENT OF NEUROLOGY
UNIVERSITY OF VIRGINIA
CHARLOTTESVILLE, VA 22908

BIELLO, KATIE BROOKS, MPH, PHD
ASSOCIATE PROFESSOR OF BEHAVIORAL AND SOCIAL SCIENCES
ASSOCIATE PROFESSOR OF EPIDEMIOLOGY
SCHOOL OF PUBLIC HEALTH
BROWN UNIVERSITY
PROVIDENCE, RI 02912

BOWMAN, NATALIE MCCARTER, MD
ASSISTANT PROFESSOR OF MEDICINE
SCHOOL OF MEDICINE
UNIVERSITY OF NORTH CAROLINA CHAPEL HILL
CHAPEL HILL, NC 27516

BROOKS, WILLIAM M, PHD
PROFESSOR
DEPARTMENT OF NEUROLOGY
DIRECTOR
HOGLUND BRAIN IMAGING CENTER
UNIVERSITY OF KANSAS MEDICAL CENTER
KANSAS CITY, KS 66160

CHEN, AIMIN, MD, PHD
PROFESSOR
DEPARTMENT OF ENVIRONMENTAL HEALTH
COLLEGE OF MEDICINE
UNIVERSITY OF CINCINNATI
CINCINNATI, OH 45267

CHEN, JIANFU, PHD
ASSISTANT PROFESSOR
CENTER FOR CRANIOFACIAL MOLECULAR BIOLOGY
UNIVERSITY OF SOUTHERN CALIFORNIA (USC)
LOS ANGELES, CA 90033

CHILIAN, WILLIAM M, PHD
PROFESSOR
DEPARTMENT OF INTEGRATIVE MEDICINE
NORTHEAST OHIO MEDICINE UNIVERSITY
COLLEGE OF MEDICINE
ROOTSTOWN, OH 44272

CHUGANI, HARRY, MD
PROFESSOR
DEPARTMENT OF NEUROLOGY
NEW YORK UNIVERSITY
LANGONE COMPREHENSIVE EPILEPSY CENTER
NEW YORK, NY 10016

CONFORTO, ADRIANA B, MD, PHD
PROFESSOR
DEPARTMENT OF NEUROLOGY
HOSPITAL DAS CLINICAS
SÃO PAULO UNIVERSITY
FUNDACAO FACULDADE DE MEDICINA
SAO PAULO,SP 01246001
BRAZIL

CORTINES, JULIANA REIS, PHD
ASSOCIATE PROFESSOR
DEPARTMENT OF VIROLOGY
MICROBIOLOGY INSTITUTE
FEDERAL UNIVERSITY OF RIO DE JANEIRO
RIO DE JANEIRO
BRAZIL

CUEVAS, JAVIER, PHD
PROFESSOR
DEPARTMENT OF MOLECULAR PHARMACOLOGY AND PHYSIOLOGY
COLLEGE OF MEDICINE
UNIVERSITY OF SOUTH FLORIDA
TAMPA, FL 33612

DHARMAKUMAR, ROHAN, PHD
PROFESSOR, BIOMEDICAL SCIENCES
CEDARS-SINAI MEDICAL CENTER
UNIVERSITY OF CALIFORNIA, LOS ANGELES
LOS ANGELES, CA 90048

ELKON, KEITH B, MBBCH, MRCP
MART MANNIK M.D.-LUCILE T. HENDERSON ENDOWED PROFESSOR
DIVISION OF RHEUMATOLOGY
UNIVERSITY OF WASHINGTON
SEATTLE, WA 98195

ERDOGMUS, DENIZ, PHD
PROFESSOR
ELECTRICAL AND COMPUTER ENGINEERING DEPARTMENT
BIOENGINEERING
NORTHEASTERN UNIVERSITY
360 HUNTINGTON AVE
BOSTON, MA 02115

FACTOR-LITVAK, PAM R, PHD
PROFESSOR AND ASSOCIATE DEAN
DEPARTMENT OF EPIDEMIOLOGY
MAILMAN SCHOOL OF PUBLIC HEALTH
COLUMBIA UNIVERSITY
NEW YORK, NY 10032

FARAH, MOHAMED HASSAN, PHD
ASSOCIATE PROFESSOR
DEPARTMENT OF NEUROLOGY
AND NEUROSCIENCE
JOHNS HOPKINS UNIVERSITY SCHOOL OF MEDICINE
JOHNS HOPKINS UNIVERSITY
BALTIMORE, MD 21205

FISHER, MARK J, MD
PROFESSOR
DEPARTMENT OF NEUROLOGY
UNIVERSITY OF CALIFORNIA IRVINE
ORANGE, CA 92868

GARCIA, FREDERICO, MD, PHD
PROFESSOR
DEPARTMENT OF MENTAL HEALTH
FEDERAL UNIVERSITY OF MINAS GERAIS
AVENIDA ALFREDO BALENA
BRAZIL

GENDELMAN, HOWARD E, MD
PROFESSOR AND CHAIR
DEPARTMENT OF PHARMACOLOGY
AND EXPERIMENTAL NEUROSCIENCE
UNIVERSITY OF NEBRASKA MEDICAL CENTER
OMAHA, NE 68198

GERAGHTY, DANIEL E, PHD
MEMBER
CLINICAL RESEARCH DIVISION
FRED HUTCHINSON CANCER RESEARCH CENTER
SEATTLE, WA 98109

HAN, RENZHI, PHD
ASSOCIATE PROFESSOR
DEPARTMENT OF SURGERY
WEXNER MEDICAL CENTER
THE OHIO STATE UNIVERSITY
COLUMBUS, OH 43210

HASCUP, ERIN RUTHERFORD, PHD
ASSOCIATE PROFESSOR
DEPARTMENT OF NEUROLOGY
CENTER FOR ALZHEIMER'S DISEASE
AND RELATED DISORDERS
SOUTHERN ILLINOIS UNIVERSITY SCHOOL OF MEDICINE
SPRINGFIELD, IL 62794

HORNER, PHILIP J, PHD
PROFESSOR AND DIRECTOR
CENTER FOR NEUROREGENERATION
CO-DIRECTOR, CTR FOR REGENE & RESTOR. NEUROSURG.
VICE CHAIRMAN, DEPARTMENT OF NEUROSURGERY
HOUSTON METHODIST RESEARCH INSTITUTE
HOUSTON, TX 77030

HOWARD, TIMOTHY D, PHD
PROFESSOR
DEPARTMENT OF BIOCHEMISTRY
CENTER FOR PRECISION MEDICINE
WAKE FOREST UNIVERSITY HEALTH SCIENCE CENTER
WINSTON-SALEM, NC 27157

HUERTA, PATRICIO T, PHD
PROFESSOR
CENTER FOR AUTOIMMUNE, MUSCULOSKELETAL AND
HEMATOPOIETIC DISEASES
FEINSTEIN INSTITUTES FOR MEDICAL RESEARCH
MANHASSET, NY 10030

JAISWAL, JYOTI K, PHD
ASSOCIATE PROFESSOR
DEPARTMENT OF GENOMICS AND PRECISION MEDICINE
GEORGE WASHINGTON UNIVERSITY SCHOOL OF MEDICINE
AND HEALTH SCIENCES
WASHINGTON, DC 20010

KUHNS, LISA MARY, MPH, PHD
RESEARCH ASSOCIATE PROFESSOR
DEPARTMENT OF PEDIATRICS
FEINBERG SCHOOL OF MEDICINE
NORTHWESTERN UNIVERSITY
CHICAGO, IL 60612

LI, JUN, MD, PHD
PROFESSOR AND CHAIRMAN
DEPARTMENT OF NEUROLOGY
WAYNE STATE UNIVERSITY SCHOOL OF MEDICINE
DETROIT, MI 48201

LUTZ, MICHAEL WILLIAM, PHD
SENIOR RESEARCH SCIENTIST
DIVISION OF NEUROLOGY
DEPARTMENT OF MEDICINE
DUKE UNIVERSITY MEDICAL CENTER
DURHAM, NC 27705

MELENDEZ, LOYDA M, PHD
PROFESSOR
DEPARTMENT OF MICROBIOLOGY
SCHOOL OF MEDICINE
UNIVERSITY OF PUERTO RICO MEDICAL SCIENCES
SAN JUAN, PR 00936

MEYER, DAWN MATHERNE, PHD
ASSOCIATE PROFESSOR
DEPARTMENT OF NEUROSCIENCES
SCHOOL OF MEDICINE
UNIVERSITY OF CALIFORNIA, SAN DIEGO
LA JOLLA, CA 92093

SCHANG, LUIS M, DVM, PHD, PDF
PROFESSOR AND DIRECTOR
BAKER INSTITUTE FOR ANIMAL HEALTH-CVN
COLLEGE OF VETERINARY MEDICINE
CORNELL UNIVERSITY
ITHACA, NY 14853

SHELTON, RICHARD CHARLES, MD
VICE CHAIR
RESEARCH AT UNIVERSITY OF ALABAMA AT BIRMINGHAM
DEPARTMENT OF PSYCHIATRY AND
BEHAVIORAL NEUROBIOLOGY
UNIVERSITY OF ALABAMA AT BIRMINGHAM
BIRMINGHAM, AL 35294

SHI, YANHONG, PHD
PROFESSOR
DEPARTMENT OF NEUROSCIENCE
BECKMAN RESEARCH INSTITUTE OF THE CITY OF HOPE
DUARTE, CA 91001

SMITH, GWENN S, PHD
PROFESSOR
DEPARTMENT OF PSYCHIATRY AND BEHAVIORAL
SCIENCES
JOHNS HOPKINS UNIVERSITY SCHOOL OF MEDICINE
BALTIMORE, MD 21224

STEIN, ARYEH DAVID, PHD
PROFESSOR
HUBERT DEPARTMENT OF GLOBAL HEALTH
ROLLINS SCHOOL OF PUBLIC HEALTH
EMORY UNIVERSITY
ATLANTA, GA 30322

STRINGER, ELIZABETH MCPHILLIPS, MD
ASSOCIATE PROFESSOR
DEPARTMENT OF OBSTETRICS AND GYNECOLOGY
UNIVERSITY OF NORTH CAROLINA
CHAPEL HILL, NC 27599-7570

VOLSKY, DAVID J, PHD
PROFESSOR
DIVISION OF INFECTIOUS DISEASES
DEPARTMENT OF MEDICINE
ICAHN SCHOOL OF MEDICINE AT MOUNT SINAI
NEW YORK, NY 10019

WALKER, CHERYL KATHERINE, MD
PROFESSOR
DEPARTMENT OF OBSTETRICS AND GYNECOLOGY
DIVISION OF MATERNAL FETAL MEDICINE
SCHOOL OF MEDICINE
UNIVERSITY OF CALIFORNIA AT DAVIS
SACRAMENTO, CA 95817

WHEELER, ANNE CAROLINE, PHD
RESEARCH PUBLIC HEALTH ANALYST
RESEARCH TRIANGLE INSTITUTE INTERNATIONAL
RESEARCH TRIANGLE PARK, NC 27709

WU, PING, MD, PHD
PROFESSOR
DEPARTMEN OF NEUROSCIENCE AND CELL BIOLOGY
UNIVERSITY OF TEXAS MEDICAL BRANCH, GALVESTON
GALVESTON, TX 77555

ZIGMOND, RICHARD E, PHD
PROFESSOR
DEPARTMENT OF NEUROSCIENCES
CASE WESTERN RESERVE UNIVERSITY
CLEVELAND, OH 44106

SCIENTIFIC REVIEW OFFICER

HODGE, DEBORAH, PHD
SCIENTIFIC REVIEW OFFICER
CENTER FOR SCIENTIFIC REVIEW
NATIONAL INSTITUTES OF HEALTH
BETHESDA, MD 20892

NADI, SUZAN, PHD
SCIENTIFIC REVIEW OFFICER
CENTER FOR SCIENTIFIC REVIEW
NATIONAL INSTITUTES OF HEALTH
BETHESDA, MD 20892

EXTRAMURAL SUPPORT ASSISTANT

PANIAGUA, MIGUEL ALEJANDRO
ADMINISTRATIVE ASSISTANT
CENTER FOR SCIENTIFIC REVIEW
NATIONAL INSTITUES OF HEALTH
BETHESDA, MD 20892

Consultants are required to absent themselves from the room during the review of any application if their presence would constitute or appear to constitute a conflict of interest.
